# KiPho: malaria parasite kinome and phosphatome portal

**DOI:** 10.1093/database/bax063

**Published:** 2017-10-10

**Authors:** Rajan Pandey, Pawan Kumar, Dinesh Gupta

**Affiliations:** 1 Translational Bioinformatics Group, International Centre for Genetic Engineering and Biotechnology, Aruna Asaf Ali Marg, New Delhi 110067, India

## Abstract

The *Plasmodium* kinases and phosphatases play an essential role in the regulation of substrate reversible-phosphorylation and overall cellular homeostasis. Reversible phosphorylation is one of the key post-translational modifications (PTMs) essential for parasite survival. Thus, a complete and comprehensive information of malarial kinases and phosphatases as a single web resource will not only aid in systematic and better understanding of the PTMs, but also facilitate efforts to look for novel drug targets for malaria. In the current work, we have developed KiPho, a comprehensive and one step web-based information resource for *Plasmodium* kinases and phosphatases. To develop KiPho, we have made use of search methods to retrieve, consolidate and integrate predicted as well as annotated information from several publically available web repositories. Additionally, we have incorporated relevant and manually curated data, which will be updated from time to time with the availability of new information. The KiPho (Malaria Parasite Kinome—Phosphatome) resource is freely available at http://bioinfo.icgeb.res.in/kipho.

## Introduction

Reversible phosphorylation is one of the key protein post-translational modifications (PTMs) involving an intricate balance between the opposing activities of kinases and phosphatases performing phosphorylation and dephosphorylation, respectively. Substrate reversible phosphorylation mostly plays a key regulatory role in protein activation and signal propagation; activating essential pathways for the growth and survival of cells (1–3). Historically, although kinases have received more attention for malaria research, however, recent studies, including the one from our laboratory, have contributed substantial data and information related to phosphatases (3–9). The information on kinases and phosphatases is increasing at such a drastic pace that even the experts find it difficult to keep track of all the publically available information (10). Additionally, kinases and phosphatases are no longer limited to a specialized research area. Scientists in general, face problem in comprehensive information retrieval regarding kinases/phosphatases. Facing similar problems in our research, we were motivated to capture the information related to kinases/phosphatases dispersed in the literature and various other sources; and develop an organized, user-friendly and search-able knowledge-base.

A number of web resources are available for specific needs in the eukaryotic kinase/phosphatase research, though none specifically for plasmodium. For example, Eukaryotic Protein Kinase and Protein Phosphatase Database, EKPD [http://ekpd.biocuckoo.org (17 August 2017, date last accessed)] maintains kinase and phosphatase information for 84 eukaryotic organisms (11). KinBase [http://www.kinase.com/kinbase/ (17 August 2017, date last accessed)] (12) and Kinweb [http://www.itb.cnr.it/kinweb/ (17 August 2017, date last accessed)] (13) maintain information related to human kinases. The web-based Kinase.com provides information related to function, evolution and diversity of protein kinases. The Protein Phosphatase Database (http://phosphatase.biochem.vt.edu) focuses on information retrieved from primary scientific literature, limiting its effort to prokaryotic phosphatases (14). PhosphaBase (http://www.bioinf.man.ac.uk/phosphabase) retrieve protein sequence information from UniProtKB (15). The Protein Tyrosine Phosphatases website [http://ptp.cshl.edu (17 August 2017, date last accessed)] provides the cellular and biological functions of protein tyrosine phosphatases, supplementing sequence and structure information (16). HuPho [http://hupho.uniroma2.it (17 August 2017, date last accessed)] (10) and DEPOD (http://depod.bioss.uni-freiburg.de) (17) contains information related to human phosphatases.

Despite availability of several resources and databases, none of the above-discussed databases is complete and provides all the information related to *Plasmodium* kinases and phosphatases on a single platform. We have developed a web resource known as ‘KiPho’ (Phospho-proteome of *Plasmodium falciparum*, *Plasmodium berghei*, *Plasmodium chabaudi* and *Plasmodium vivax*), with an aim to provide information related to *Plasmodium* kinases and phosphatases to the researchers interested in *Plasmodium* genes, as well as those who just want to rapidly retrieve information for particular kinases or phosphatases. KiPho is freely accessible at http://bioinfo.icgeb.res.in/kipho (17 August 2017, date last accessed).

KiPho provides a user-friendly and searchable platform for retrieval of information regarding structure similarity, function and published literature about *Plasmodium* kinases and phosphatases. Most of the information in this web resource is dynamically retrieved from publicly available repositories, which are integrated with data curated by us in the KiPho internal database.

## Materials and methods

### Identification, annotation and manual curation of the kinases and phosphatases

**Figure 1.**
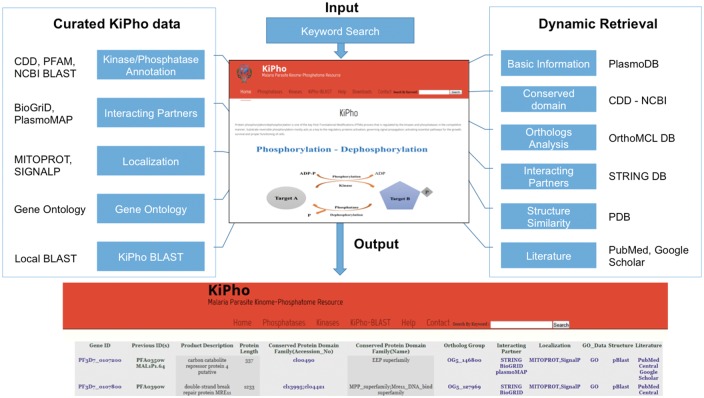
The schema for KiPho development and operational workflow.

The schema for KiPho development and workflow is shown in Figure 1. Proteomes of *Plasmodium* sps. *P. falciparum*, *P. berghei*, *P. chabaudi* and *P. vivax* were retrieved from PlasmoDB (v 9.2). CDD and PFAM search was performed for the retrieved proteins for its classification into kinase and phosphatase families based on conserved domains present in the sequences. KiPho uses BioGrid, plasmoMAP and STRING predictions to obtain predicted interacting partners. STRING database is dynamically linked whereas BioGrid and plasmoMAP are maintained and updated internally. MitoProt is used for mitochondrial target gene identification (24). SignalP 4.1 server was used for the signal peptide and transmembrane sequence identification in query proteins (25).

### KiPho web portal

The KiPho web interface has been designed in HTML5, CSS and JavaScript languages with PHPver5.2 running the queries. CSS and JavaScript encode the interactive graphical user interface for the web pages. Tab-separated values (TSV) files are used for storing data. KiPho also link the web portal to other data sources; PlasmoDB, STRING, PUBMED, Google Scholar, OrthoMCL, CDD and NCBI BLASTP to retrieve related useful information dynamically. The custom search terms for each class of phosphatases and kinases ensure that no related entry is missed due to the ambiguity of nomenclatures.

### Incorporated tools in the web portal

KiPho integrates Basic Local Alignment Search Tool (BLAST) version 2.2.28 from NCBI to perform local BLAST. The makeblastdb program was used to convert the FASTA-formatted local file (files having kinase and phosphatase sequences, extracted from KiPho database) into a BLAST database and perform blast operations. BioPython ver 1.68 scripts were used to link and perform NCBI BLAST, which displays best five hits.

## Results and discussion

The KiPho project addresses the need for information retrieval for *Plasmodium* kinases and phosphatases. The KiPho database development started with retrieval and classification of kinases and phosphatases on the basis of intrinsic conserved domains responsible for respective functions. We identified relevant web resources; PlasmoDB (*Plasmodium* Genomic Resource), OrthoMCL (Ortholog Groups of Protein Sequences), Gene Ontology (Gene Function annotation database), PUBMED and GOOGLE SCHOLAR for related literature informations, NCBI BLAST to find related query sequences, and PlasmoMAP (Functional interaction of *P. falciparum*), BioGrid (General repository for interaction datasets) and STRING database for protein–protein interactions, which provide necessary information for *Plasmodium* kinases and phosphatases. We used bioinformatics tools, which include CDD, PFAM, MitoProt, NCBI BLASTp, SignalP and BioPython to predict structural and functional role of the proteins and to provide additional information, which were surprisingly missing in several cases. Finally, we used key-word searches like old and new PlasmoDB IDs and its name to identify literature (research and review articles) using text mining. This resulted in a comprehensive resource for extracting any published information for plasmodium kinases and phosphatases.

Despite the best of our effort, it cannot be claimed that KiPho is a complete web resource for *Plasmodium* kinases/phosphatases due to research advancement and the inclusion of new features and functions assigned to these genes. However, the website is designed to facilitate new annotations, retrieve information dynamically from linked databases and will be updated from time to time. Also, users and experts in malaria research are encouraged to contact authors for any discrepancies or point out any missing information, which can be incorporated into the database. 

### Protein kinase and phosphatase classification

We retrieved *P. falciparum* (5712), *P. berghei* (5246), *P. chabaudi* (5364) and *P. vivax* (5631) annotated protein sequences from PlasmoDB version 9.2 (18). We performed CDD (19) and PFAM (20) searches to identify phosphatase and kinase domain-containing proteins in the retrieved sequences. This resulted in the identification of 277 *Plasmodium* phosphatase proteins, out of which, 70 *P. falciparum*, *67 P. berghei*, *67 P. chabaudi* and *71 P. vivax* sequences were found. The search also yielded 524 *Plasmodium* kinase proteins, out of which, 147 *P. falciparum*, 123 *P. berghei*, 124 *P. chabaudi* and 130 *P. vivax* sequences were found. The proteins represent 13 phosphatase and 16 kinase superfamilies.

### KiPho database GUI

**Figure 2.**
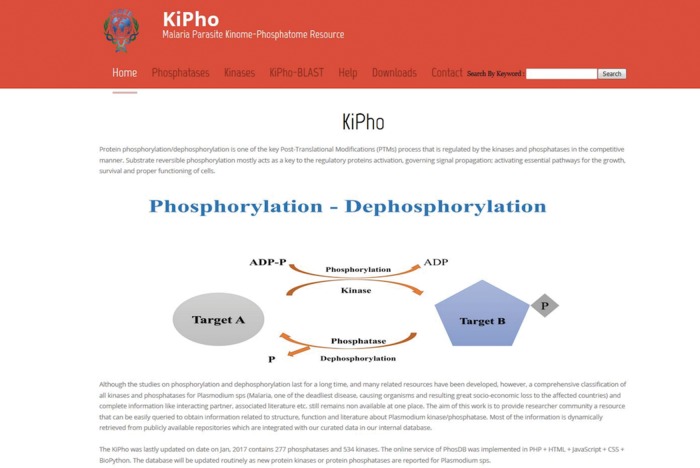
KiPho web-based GUI interface. The database may be queried in different ways.

KiPho serves as web portal as well as a database of kinases and phosphatases found in selected *Plasmodium* species (Figure 2). KiPho offers two types of easy search options: a keyword search field and descriptive kinase/phosphatase tab option which displays a complete list of *P. falciparum*, *P. berghei*, *P. chabaudi* and *P. vivax* kinases/phosphatases with relevant details and links.

By using the search tab option, a search can be performed by typing old or new PlasmoDB IDs, UniProtKB ID, Entrez Gene ID, product name or keywords like protein name, function, etc. KiPho retrieves the protein matching the description with user-entered string and displays it. The protein of interest can be picked from the output. Once a protein is selected, all the information related to the protein can be retrieved from curated tables and linked publically available web sources (PlasmoDB, STRING and OrthoMCL), organized as various tabs.

### Description tab

This tab provides basic information for query kinases and phosphatases including protein name, present and previous PlasmoDB ID, gene name, UniProtKB ID, Entrez Gene ID as retrieved from PlasmoDB together with superfamily classification based on PFAM and CDD. This page also provides links for other details like PDB BLAST, subcellular localization or interacting partner tab. PDB BLAST helps in identification of similar protein sequences for which experimental crystal/NMR structure has been solved and may be used as a template for prediction of three-dimensional structure for the query proteins to which no experimental 3D structure is available. MitoProt was used to identify mitochondrial targeted sequences. MitoProt uses N-terminal protein sequence region that can support a mitochondrial targeted sequence and cleavage site. We used STRING, PlasmoMAP and BioGrid to predict interacting partners for the queried proteins. The analysis may help in the identification and functional characterization of query kinase and phosphatase proteins, based on interacting partners and prediction of pathways involved.

### Protein interaction tab

Protein–protein interactions involved in cellular processes are an important aspect of studying functions performed by proteins. It also indicates the importance of the normal proteins function for cell and organisms survival. BioGrid (21) is a curated interaction repository. PKs and PPs interactors shown in BioGrid are based on two-hybrid physical interaction evidences. PlasmoMAP (22) is the functional interactome of *P. falciparum* and the interaction network is predicted by integrating computational and functional genomic data. We have included only the high confidence PlasmoMAP interactions for the inclusion into KiPho. STRING database provides information regarding known and predicted protein–protein interactions (23). STRING includes protein–protein interaction information from experimentally derived interactions through literature curation, text mining and interaction interfered from model organisms based on orthology, interactions computed from genomic features, co-expression, neighborhood and co-occurrence. For KiPho, we have used dynamic version of STRING and an in-house curated PlasmoMAP, and BioGrid dataset of *P. falciparum*. Using KiPho, one may retrieve and compare results at the same time.

### Structure homology, gene ontology, orthologs and literature tab

KiPho uses a BioPython script to retrieve five best sequences of PDB structures from the PDB database, with significant similarity to the query proteins, if available. This information can be used to predict homology-based or *ab initio* 3D models for a query protein, in case a crystal/NMR structure is not available. In the next update, we will incorporate repository of predicted 3D models using homology methods for proteins for which no crystal/NMR structure is available. Orthologs tab contains information related to paralogs and orthologs of the query kinase/phosphatase. Orthologs tab dynamically retrieves orthologs from the updated current version of OrthoMCL database to display orthologs of query proteins.

KiPho provides PubMed link and google scholar link for research and review articles related to the query proteins, published till date. KiPho uses scripts, which in turn uses Boolean logic search that includes its name, protein, new and old IDs assigned to it. This method helps the user to find all updated query protein-related publications.

## Authors’ contributions

R.P. performed database, literature searches, sequence analysis and annotations. P.K. developed the background scripts. P.K. and R.P. designed the database and the website. R.P. and D.G. performed the analysis and wrote the manuscript. D.G. and R.P. formulated the research idea and KiPho schema. D.G. supervised the study. All authors read and approved the manuscript.

## Funding

Department of Biotechnology, Govt. of India (BT/PR6963/BID/7/427/2012, BT/BI/25/066/2012) awarded to D.G.

Conflict of interest: None declared.
